# Persistent Raynaud's Phenomenon Following Methylphenidate Hydrochloride Use During the COVID-19 Pandemic

**DOI:** 10.7759/cureus.17647

**Published:** 2021-09-01

**Authors:** Christopher Laboe, Emma Batchelder, Deepa Vasireddy

**Affiliations:** 1 Psychiatry, Penn State College of Medicine, Hershey, USA; 2 Psychiatry, University of Virginia, Charlottesville, USA; 3 Pediatrics, Pediatric Group of Acadiana, Lafayette, USA

**Keywords:** raynaud’s phenomenon, methylphenidate hydrochloride, covid-19, stimulant, adhd, suicide and depression

## Abstract

Raynaud's phenomenon (RP) is a medical condition characterized by vasospasm of the digital vessels in the fingers and toes. The prevalence of RP in the general population is estimated at 3-5% and can vary based on climate. It is classified into primary and secondary RP based on causality. RP has been reported in some cases diagnosed with coronavirus disease 2019 (COVID-19) infection. We report the case of a 14-year-old Caucasian female who presented during the pandemic with chief complaints of suicidal ideations and attempted suicide and had a history of attention-deficit hyperactivity disorder (ADHD) and persistent RP after a stimulant trial. After an initial failure of treatment with lisdexamfetamine, she was switched to methylphenidate hydrochloride (MPH). Within two months of starting MPH, the patient noticed skin discoloration of the lower legs and feet along with numbness. The discoloration of skin was mainly limited to her feet and gradually moved up her legs. She was advised to discontinue the MPH, but her symptoms persisted for four more months until her admission. Other etiologies were ruled out by multi-specialties and during her hospitalization. She was started on atomoxetine and buspirone with appropriate dose titration. Post-discharge from the hospital, no improvement was observed in the patient's RP at an outpatient follow-up performed within a month. The development of RP following MPH treatment and its persistence after stopping MPH is a fascinating event. Clinicians should be aware of the potential rare side effects of stimulants and stimulant-like medications, including vascular, hematological, and dermatological effects. Adolescents with ADHD may be particularly distressed by the COVID-19 pandemic and display increased behavioral issues. Stress can be a trigger for RP; therefore, minimizing stress in at-risk patients is essential.

## Introduction

Raynaud's phenomenon (RP) is a medical condition characterized by vasospasm of the digital vessels in the fingers and toes. RP exacerbation occurs by cold, stress, and medications, resulting in changes in the color of the digits, ranging from white to blue to red [1]. The blanching is a result of vasoconstriction/occlusion of precapillary arterioles. Cyanosis occurs due to the deoxygenation of trapped blood in post-capillary venules. Rubor manifests when the ischemia resolves, leading to hyperemia [1]. The prevalence of RP in the general population is estimated at 3-5% and can vary based on climate [2]. An unidentified trigger can lead to a condition known as primary RP. If there is an attributable cause, such as a disease or medication, then the condition is known as secondary RP [1]. Secondary causes include connective tissue diseases and drugs, such as non-selective beta-blockers and stimulants [3]. RP has been reported in some cases diagnosed with coronavirus disease 2019 (COVID-19) infection [4]. It is noteworthy that medical and psychiatric illnesses reported during the COVID-19 pandemic have had varied and unprecedented types of presentations. Numerous studies have documented exacerbation and different manifestations of diseases either due to pandemic-related stress or due to infection and post-infection sequelae [2,5,6]. In this report, we discuss the case of a 14-year-old Caucasian female who presented with chief complaints of suicidal ideations and attempted suicide by overdosing on ibuprofen tablets and had a history of attention-deficit hyperactivity disorder (ADHD) and persistent RP after a stimulant trial. The patient’s identity has been kept confidential in the manuscript.

## Case presentation

The patient was a 14-year-old Caucasian female with a psychiatric history of ADHD and generalized anxiety disorder and no other past medical history. She presented in August 2020 during the COVID-19 pandemic with worsening depression, anxiety, and inattention, following the exacerbation of suicidal ideations secondary to pandemic-related stress and isolation, which had led her to take a handful of ibuprofen pills. The COVID-19 pandemic was described by the patient as a huge stressor, leading to worsening depression and anxiety. At admission, the patient was negative for COVID-19 when tested as part of the initial screening. After being medically cleared, she was admitted to inpatient psychiatry. She had no other past medical, allergic, and surgical history and no history of psychiatric or medical hospitalization. The patient was not on any medications at the time of admission. Baseline investigations including EKG, complete blood count, comprehensive metabolic panel, and thyroid panel were within normal limits.

**Figure 1:**
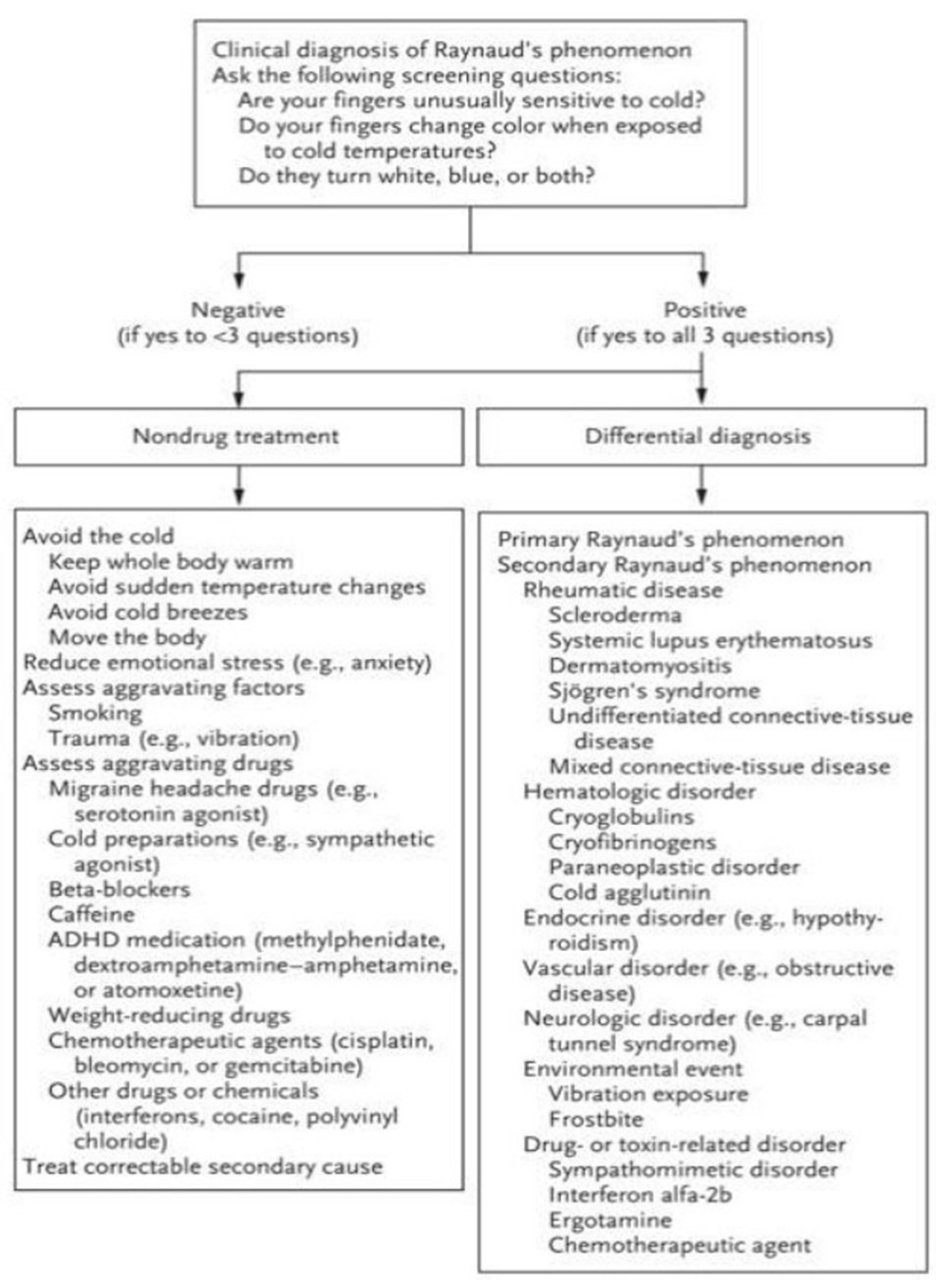
Differential diagnosis and nonpharmacological treatment of Raynaud's phenomenon* *[7] ADHD: attention-deficit hyperactivity disorder

On admission, a diagnosis of major depressive disorder (MDD), first episode, severe and ADHD-inattentive type was established. The patient had been diagnosed with ADHD approximately a year ago and started on a trial of lisdexamfetamine. The lisdexamfetamine had been initiated at a dose of 10 mg/day and titrated up to 30 mg/day within a month, and she had continued with this dosage for two months. She had experienced no side effects due to lisdexamfetamine. The patient and her parents had felt that the lisdexamfetamine was not helping with her inattention, and after two months, she had discontinued lisdexamfetamine and switched to methylphenidate hydrochloride (MPH). About six months before the admission, MPH had been initiated and titrated to 60 mg/day within one month. Within two months of starting MPH, the patient had noticed skin discoloration of the lower legs and feet. She had been evaluated by Cardiology and Psychiatry on an outpatient basis and advised to stop MPH. The patient had continued to have waxing and waning of lower leg discoloration that had been nontender and blanched upon palpation for four months until her admission. Her symptoms occurred approximately once per day, usually in the afternoon and with varying duration. Discoloration of the skin had been mainly limited to her feet and had gradually moved up her legs. She believed the discoloration worsened with exposure to cold and was not associated with stress. She experienced numbness of her lower legs and feet but no other accompanying symptoms with the discoloration. She did not have a history of substance use or smoking. Given the multitude of causes of RP, we enquired about any family history of RP, autoimmune diseases, connective tissue diseases, rheumatological, cardiovascular, or neurological diseases, which was negative (Figure 1).

According to a review of the patient's prior history, she had not had a COVID-19 test prior to this admission and the COVID-19 symptom list had been negative within six months leading up to the admission. Around the time of admission, she had a cardiology follow-up with EKG, ultrasound with Doppler of the lower legs, and echocardiography, which were all negative. She had normal blood chemistry and hematology results ruling out other causes of RP. The patient did not have a history of previous use of other psychotropic medications before her admission. During her hospitalization, she was started on atomoxetine 10 mg daily to treat her depression and inattention and the dose was gradually titrated up to 40 mg. Atomoxetine's action as a selective norepinephrine reuptake inhibitor suggested its efficacy as an antidepressant and it was deemed an appropriate option for the patient. She responded well to this medication with no side effects. She was also started on buspirone and titrated up to 10 mg twice daily for her anxiety symptoms. The patient did well with cognitive behavioral therapy skills and other behavioral modification and relaxation techniques that she learned while in the inpatient unit. Post-discharge from the hospital, it was found that there was no improvement in the patient's RP at an outpatient follow-up conducted within a month. The patient continued to remain compliant with her outpatient psychiatry and cardiology appointments.

## Discussion

It appears that psychostimulants reduce the diameter of the vasculature, which can create filling defects, thereby leading to hypertension [8]. Changes in blood pressure can lead to changes in shearing forces that could damage blood vessels. A case series looking at stimulants leading to RP in adolescents found elevated anti-histone antibodies to measure drug-induced vasculopathy. It was still unclear if the stimulants were a direct cause of the RP or other associated factors [9]. The development of RP following MPH treatment and its persistence after the discontinuation of MPH is a fascinating event. Although the pathogenesis of RP is not well understood, one of the proposed mechanisms is that excess catecholamine release results in a net vasoconstrictive effect [10]. MPH causes a release of catecholamines due to its impact on dopaminergic and noradrenergic systems [11]. Dose-related RP effects while on MPH have been previously reported [10]. The authors were able to identify only one published case series within the past decade describing the relationship between MPH and RP [3]. Another distinct possibility is a latent COVID-19 infection or exacerbation of symptoms of RP secondary to increased anxiety and depression caused by the sense of isolation due to the pandemic [5,12,13]. A previously documented case describes a 19-year-old female who initially developed mild RP that worsened in severity over three years while on MPH and was subsequently diagnosed with systemic sclerosis [3]. Upon discontinuation of the MPH, there was no improvement in her RP symptoms.

In our patient, the reported discoloration and numbness of feet and lower legs started two months after initiating an MPH trial; however, the same effects did not occur with a two-month trial of lisdexamfetamine. Another interesting observation was the persistence of RP despite stopping the offending agent, which has not been previously reported in the case of stimulants causing RP. One explanation of our patient's uniform, bilateral lower leg, and foot discoloration could be that alpha-2 receptors are more prominent on the distal arteries [14]. It is possible that increased sympathetic noradrenergic nerve activity in response to cold or mental stress occurred at the time of MPH use, in contrast with the lisdexamfetamine trial, resulting in the presence of uniform, bilateral lower legs and feet discoloration [9,14]. Clinicians should be aware of the rare but potential side effects of stimulants and stimulant-like medications, including vascular, hematological, and dermatological effects when selecting medications and considering the side effect profiles of individual drugs [15,16]. RP is associated with other vasoconstrictive medications, including beta-blockers, clonidine, ergot alkaloids, dopaminergic agonists, selective serotonin reuptake inhibitors, cyclosporin, cocaine, and various cancer chemotherapies [17]. The synergistic impact of RP risk and stimulant medication with other RP-associated therapies should be considered when initiating treatment for ADHD. A primary care survey performed during the COVID-19 pandemic measuring depression and suicide risk in adolescents aged 12 to 21 years showed that depression and suicide concerns have increased among adolescents, particularly female adolescents [18]. Adolescents with ADHD may be particularly distressed by the COVID-19 pandemic and display increased behavioral issues [19]. Particular emphasis needs to be placed on increasing social connectivity for teenagers and young adults in these times of enforced social isolation [20].

## Conclusions

Clinician anticipation and awareness of RP as a potential side effect of MPH may lead to earlier recognition and discontinuation of the medication, thereby reducing this effect. It is of paramount importance to perform more studies to identify specific biological and environmental factors leading to the presentation of RP as a result of psychostimulant use in adolescents and young adults. Clinicians need to be mindful of the extreme stress such patients are under, especially secondary to the social isolation in the times of the COVID-19 pandemic. Stress can be a trigger for RP; therefore, minimizing stress in at-risk patients is essential.
